# Immunomics in one health: understanding the human, animal, and environmental aspects of COVID-19

**DOI:** 10.3389/fimmu.2024.1450380

**Published:** 2024-09-04

**Authors:** Jing Gao, Chutian Zhang, Åsa M. Wheelock, Siming Xin, Hui Cai, Lei Xu, Xiao-jun Wang

**Affiliations:** ^1^ Department of Respiratory Medicine, Gansu Provincial Hospital, Lanzhou, China; ^2^ Respiratory Medicine Unit, Department of Medicine Solna, Center for Molecular Medicine, Karolinska Institutet, Stockholm, Sweden; ^3^ Department of Pulmonary Diseases, University of Helsinki and Helsinki University Hospital, Helsinki, Finland; ^4^ Vanke School of Public Health, Tsinghua University, Beijing, China; ^5^ College of Natural Resources and Environment, Northwest Agriculture and Forestry University, Yangling, China; ^6^ Department of Respiratory Medicine and Allergy, Karolinska University Hospital, Stockholm, Sweden; ^7^ The First School of Clinical Medicine, Gansu University of Chinese Medicine, Lanzhou, China; ^8^ Department of General Surgery, Gansu Provincial Hospital, Lanzhou, China; ^9^ Institute for Healthy China, Tsinghua University, Beijing, China

**Keywords:** immunomics, one health, SARS-CoV2, COVID-19, immunology

## Abstract

The coronavirus disease 2019 (COVID-19) pandemic underscores the critical need to integrate immunomics within the One Health framework to effectively address zoonotic diseases across humans, animals, and environments. Employing advanced high-throughput technologies, this interdisciplinary approach reveals the complex immunological interactions among these systems, enhancing our understanding of immune responses and yielding vital insights into the mechanisms that influence viral spread and host susceptibility. Significant advancements in immunomics have accelerated vaccine development, improved viral mutation tracking, and broadened our comprehension of immune pathways in zoonotic transmissions. This review highlights the role of animals, not merely as carriers or reservoirs, but as essential elements of ecological networks that profoundly influence viral epidemiology. Furthermore, we explore how environmental factors shape immune response patterns across species, influencing viral persistence and spillover risks. Moreover, case studies demonstrating the integration of immunogenomic data within the One Health framework for COVID-19 are discussed, outlining its implications for future research. However, linking humans, animals, and the environment through immunogenomics remains challenging, including the complex management of vast amounts of data and issues of scalability. Despite challenges, integrating immunomics data within the One Health framework significantly enhances our strategies and responses to zoonotic diseases and pandemic threats, marking a crucial direction for future public health breakthroughs.

## 1 Introduction

Immunomics, an interdisciplinary field, is essential for understanding the regulation and response of the immune system to pathogens. By integrating disciplines such as genomics, transcriptomics, proteomics, and bioinformatics, it offers a comprehensive view of the complex mechanisms that govern immune responses (1). Genomics plays a crucial role in identifying genetic variations that dictate individual responses to diseases and vaccines, thereby enhancing our understanding of host susceptibility and immune functionality (2). Transcriptomics delves into the gene expression patterns activated during immune responses, particularly those triggered by pathogens such as of severe acute respiratory syndrome coronavirus 2 (SARS-CoV-2), to reveal cellular adaptive strategies in the face of infections (3). Meanwhile, proteomics focuses on profiling critical proteins involved in immune signaling, including cytokines and chemokines, which are central to the orchestration of immune responses (4). Bioinformatics synthesizes these diverse datasets, enabling complex analyses that forecast disease progression, tailor treatments, and facilitate the development of targeted vaccines and immunotherapies (5). High-throughput techniques such as next-generation sequencing and proteomics allow immunomics to provide a comprehensive understanding of the molecular mechanisms driving immune responses, aiding the development of innovative immunotherapies, vaccines, and diagnostic tools. This integrative science is crucial for elucidating how organisms defend against pathogens at a molecular level, thereby improving our knowledge of host defense mechanisms against complex diseases like coronavirus disease 2019 (COVID-19) (5).

Leveraging the comprehensive insights provided by immunomics, the One Health approach emphasizes the interconnectedness of human, animal, and environmental health, enhancing our ability to address global health threats through a collaborative strategy (6). Immunomics, strategically positioned at the nexus of COVID-19 research and the One Health approach, employs advanced omics technologies to probe the immune system’s intricate molecular responses. This approach highlights the connections between human, animal, and environmental health. Cross-species immune research provides insights into how COVID-19 and similar pathogens interact with various biological systems. Studying immune responses in species such as mink, bats, and felines helps understand SARS-CoV-2’s evolution and spread, which can aid in preventing cross-species transmission (7–9). Environmental factors also influence viral transmission, with the One Health framework examining how climate change and habitat destruction affect host immune systems and contribute to zoonotic disease risks (10, 11). Global surveillance systems that integrate data from human, animal, and environmental health are important for early detection and containment of outbreaks (12). Advanced techniques like multi-omics and single-cell analysis facilitate the identification of biomarkers and tracking of environmental vectors to manage viral activity and transmission (7, 13, 14). Vaccine development should consider the need for vaccines that generate broad immune responses across species to help reduce outbreak risks (15, 16). The One Health framework also emphasizes the value of a coordinated global response (12).

Despite the potential benefits of integrating immunology with the One Health framework to combat COVID-19, several notable shortcomings in current research need to be addressed. Firstly, data collection on immune responses across species is fragmented and sparse, necessitating systematic and comprehensive global databases to better understand cross-species interactions with SARS-CoV-2 and prevent transmission events. Current research often segments human immunological responses from animal and environmental studies, leading to a fragmented understanding of pathogen behavior across different hosts and environments. Secondly, studies on environmental impacts like climate change and habitat destruction lack depth, requiring longitudinal research and the use of satellite data and artificial intelligence (AI) to model these effects on viral dynamics and host immune responses. Thirdly, global surveillance systems are often siloed and insufficiently integrated, highlighting the need for a unified, real-time network using advanced techniques like multi-omics and blockchain technology to enhance data integrity and sharing. Additionally, vaccine development has focused too narrowly on humans, overlooking the benefits of multi-species vaccines designed through synthetic biology and cross-species trials to reduce virus reservoirs. There is also a lack of a cohesive global response framework that integrates human, animal, and environmental health measures. Lastly, the underutilization of advanced immunomic technologies like next-generation sequencing (NGS) and Cytometry by Time-of-Flight (CyTOF) emphasizes the need for greater international collaboration to advance immune response research and targeted therapies.

**Figure 1:**
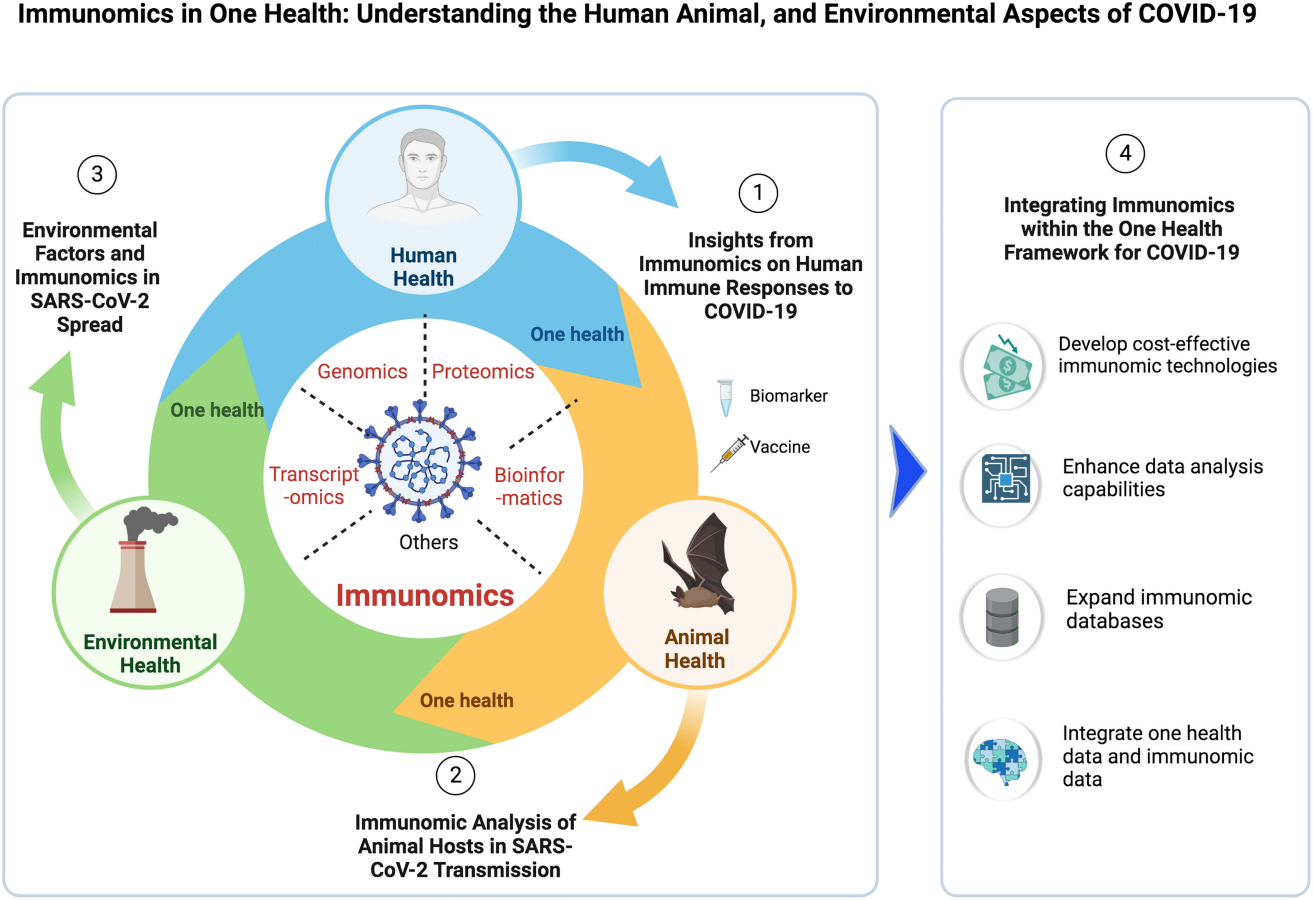
Immunomics in the One Health Framework for COVID-19 Study Overview. COVID-19, coronavirus disease 2019; SARS-CoV-2, severe acute respiratory syndrome coronavirus 2. Created using BioRender.com.

This review is organized into four key sections, each addressing a crucial aspect of immunomics within the One Health framework for COVID-19 research (**Table 1**, **Figure 1**; **Supplementary Table 1**). The sections are as follows: “ Insights from Immunomics on Human Immune Responses to COVID-19,” which examines the varied immune responses in humans; “Immunomic Analysis of Animal Hosts in SARS-CoV-2 Transmission” which investigates animal contributions to the disease’s spread; “ Environmental Factors and Immunomics in SARS-CoV-2 Spread,” which assesses how the environment facilitates the virus’s spread; “ Integrating Immunomics within the One Health Framework for COVID-19,” which offers a holistic view of pandemic dynamics within global health ecosystems; and “Methodological Strategies for Data Collection,” which focuses on the methods used to gather immunomics data and highlights the practical aspects of these approaches. This review broadens our understanding of how COVID-19 impacts humans, animals, and the environment, potentially strengthening our global response to pandemic threats.

**Table 1:** Summary of Primary Biospecimen Types, Omics Data, and High-Throughput Techniques from Immunology and One-Health Publications.

Authors	PMID	Type	Biospecimen type	Immunology	Omics	High-throughput techniques	Other	One health
Oreshkova N, et al. (7)	32553059	Environmental, Human, Animal	Lung specimens	–	Genomics, Bioinformatics	NGS	–	Yes
Zhou P, et al. (8)	32015507	Human, Animal	Oral swabs, anal swabs, blood and BALF samples,	–	Genomics, Bioinformatics	NGS	–	Yes
Halfmann PJ, et al. (9)	32402157	Human, Animal	Nasal and rectal swabs	–	–	–	–	Yes
Rüegg SR, et al. (12)	28261580	Environmental, Human, Animal	–	–	–	–	–	Yes
Liao M, et al. (13)	32398875	Human	BALF mmune cells	Proinflammatory monocyte-derived macrophages	Transcriptomics	scRNA-seq	–	No
Unterman A, et al. (14)	35064122	Human	PBMCs	Immune cells	Proteomics, Transcriptomics, Bioinformatics	Single-cell multi-omics	–	No
Ivanova EN, et al. (15)	38213787	Human	PBMCs	Cytotoxic gene and immune cells	Proteomics	scRNA-seq	–	No
Munster VJ, et al. (16)	32396922	Animal	Blood,nose swab	Animal model of COVID-19	–	–	–	No
Lucas C, et al. (17)	32717743	Human	PBMCs	cytokines and immune cells	–	–	–	No
Mathew D, et al. (18)	32669297	Human	PBMCs	Immune perturbations	–	–	High-dimensional flow cytometry	No
Penttilä PA, et al. (19)	33715015	Human	WB samples	Immunomodulatory effects	–	–	CyTOF	No
Rendeiro AF, et al. (20)	33361110	Human	PBMCs	Immune cells	–	–	Flow cytometry	No
Zhao XN, et al. (21)	34531370	Human	PBMCs	Immune cells behave	Transcriptomics	TCR/BCR sequencing	–	No
Berentschot JC, et al. (22)	37881427	Human	PBMCs	Immunological profiling	–	–	Flow cytometry	No
Mehta P, et al. (24)	32192578	Human	–	Cytokines	–	–	–	No
Huang C, et al. (25)	31986264	Human	–	Cytokines	–	–	real-time RT-PCR and NGS	No
Sekine T, et al. (26)	32979941	Human	PBMCs	Memory T cells	–	–	Flow cytometry	No
Diao B, et al. (27)	32425950	Human	PBMCs	T cells and cytokine	–	–	Flow cytometry	No
Woodruff MC, et al. (28)	33028979	Human	PBMCs	B cell responses	–	–	High-dimensional flow cytometry	No
Kuri-Cervantes L, et al. (31)	32669287	Human	Whole blood	Immune cell subsets	–	–	Flow cytometry	No
Li S,et al. (32)	33717140		PBMCs	Immune cell phenotype	Transcriptomics	scATAC-seq, scRNA-seq	–	No
Wilk AJ, et al. (33)	32514174	Human	PBMCs	Immune cell phenotype	Transcriptomics	sscRNA-seq	–	No
Zhang Q. et al. (36)	32972995	Human	Whole blood	Immunity	Genomics	NGS	–	No
Ellinghaus D, et al. (37)	32558485	Human	Whole-blood samples	Genetic factors	Bioinformatics	–	–	No
Wu H, et al. (38)	35022412	Human	Lung specimens	Immune cells	Transcriptomics	Bulk RNA sequencing and digital spatial profiling (DSP)	–	No
Herold T, et al. (39)	32425269	Human	–	Cytokines	–	–	–	No
Keech C, et al. (42)	32877576	Human	Blood	Immune cells	–	–	ELISA	No
Logunov DY, et al. (45)	32896291	Human	Blood and urine	Immune cells	–	–	Flow cytometry	No
Khoury DS, et al. (46)	34002089	Human	–	Neutralizing antibody levels	Bioinformatics	–	–	No
Korber B, et al. (49)	32697968	Human	Nose/throat swabs	Tracking Changes in SARS-CoV-2 Spike	Genomics, Bioinformatics	–	–	Yes
Walls AC, et al. (51)	32155444	Animal	–	Structure, Function, and Antigenicity of the SARS-CoV-2 Spike Glycoprotein	Bioinformatics	–	–	Yes
Gottlieb RL, et al. (54)	33475701	Human	–	–	–	–	–	No
Zhou Q, et al. (56)	32574262	Human	Serum	Cytokines	–	–	–	No
Huaman MA, et al. (57)	37534607	Human	–	–	–	–	–	No
Lam TT, et al. (60)	32218527	Animal	–	–	Genomics, Bioinformatics	RNA sequencing	–	Yes
Shi J, et al. (64)	32269068	Environmental, Human, Animal	Nasal washes and rectal swabs	–	–	–	RT-PCR	Yes
Palmer MV, et al. (65)	33692203	Animal	Nasal secretions, feces, serum tracheal wash, and lung lavage	–	Bioinformatics	–	–	No
Wan Y, et al. (66)	31996437	Human, Animal	–	Receptor recognition mechanisms	Bioinformatics	–	–	Yes
Andersen KG, et al. (70)	32284615	Human, Animal	–	The origin of SARS-CoV-2	Genomics, Bioinformatics	–	–	Yes
Kim YI, et al. (72)	32259477	Animal	Nasal washes, saliva, urine, and feces	Animal model of COVID-19	–	–	–	No
Plante JA, et al. (97)	33106671	Human, Animal	Cell lines	The D614G mutation in the USA-WA1/2020 strain	Bioinformatics	Sanger sequencing	–	No

NGS, Next Generation Sequencing; scRNA-seq, Single-cell RNA Sequencing; scATAC-seq, Single-cell Assay for Transposase-Accessible Chromatin using sequencing; TCR/BCR sequencing, T-cell/B-cell Receptor Sequencing; BALF, Bronchoalveolar Lavage Fluid; PBMCs, Peripheral Blood Mononuclear Cells; CyTOF, High-dimensional cytometry by time-of-flight; ELISA, Enzyme-Linked Immunosorbent Assay.

## 2 Insights from immunomics on human immune responses to COVID-19

### 2.1 Overview of immune responses in humans

Immunomics has significantly advanced our understanding of the heterogeneous immune responses to COVID-19, which range from mild to severe or fatal outcomes (1, 17). SARS-CoV-2 triggers complex immune responses, categorized into innate and adaptive immunity. The innate immune system, as the first line of defense, includes physical barriers and cellular responses from natural killer (NK) cells, macrophages, and dendritic cells. These responses are rapid and non-specific, acting without prior exposure. In contrast, the adaptive immune system, involving B cells and T cells, provides specific and long-lasting immunity. B cells produce antibodies to neutralize the virus, while T cells eliminate infected cells and support other immune components, establishing immunological memory for future protection. Research has significantly advanced our understanding of these immune responses in COVID-19. Notable research by Mathew et al. (18) identified three distinct immunotypes of lymphocyte responses correlated with COVID-19 severity in hospitalized patients. The first immunotype, associated with severe disease, is characterized by strong CD4 T cell activation, reduced follicular helper cells, and exhausted CD8 T cells. The second immunotype, linked to less severe disease, features reduced CD4 T cell activation and an increase in memory B cells. The third immunotype displays minimal activation of both T and B cells and is inversely related to disease severity. In critically ill patients, recovery began with classical monocytes, followed by CD8+ and CD4+ T cells, and then non-classical monocytes (19). Rendeiro et al. (20) documented dynamic alterations in immune landscapes during COVID-19 progression, highlighting significant differences among severe, mild, and healthy controls. Severe outcomes were associated with specific NK cell KIR receptor usage and IgM+ B cells, coupled with CD4 and CD8 T cell exhaustion. Additionally, Zhao et al. (21) identified a distinct immune profile in asymptomatic patients, marked by an increase in CD56+CD16- NK cells and elevated levels of interferon-gamma. These patients exhibited significant T-cell receptor (TCR) clonal expansion, especially in effector CD4+ T cells, whereas patients with moderate symptoms showed more modest B-cell receptor (BCR) clonal expansion. Asymptomatic individuals also had lower expression of interferon-stimulated genes (ISGs), though this varied considerably among patients. This profile suggests a unique immune response that may contribute to the asymptomatic presentation of the disease. Berentschot et al. (22) found that in long COVID patients, increased fatigue severity is associated with heightened monocyte activation, low-grade inflammation, and T-lymphocyte senescence. Cytokines, crucial for immune signaling, play a pivotal role in the body’s reaction to SARS-CoV-2. A “cytokine storm”, an excessive cytokine response, is significant in severe COVID-19 cases (23, 24), leading to severe inflammation and tissue damage. Elevated cytokine levels, including interleukin-6 (IL-6) and tumor necrosis factor-alpha (TNF-a), correlate with adverse outcomes (25). T-cell responses are critical for viral control and long-term immunity. Sekine et al. (26) demonstrated robust memory T-cell responses in convalescent individuals, while severe cases often show T-cell exhaustion, reducing response efficacy (27). B cells, through virus-specific antibodies, are essential for controlling SARS-CoV-2. Longitudinal studies show varying antibody durability, but memory B cells persist, suggesting sustained immune protection (28). Extrafollicular B cell activation was strongly correlated with extensive expansion of antibody-secreting cells and early production of high concentrations of SARS-CoV-2-specific neutralizing antibodies (28).These studies collectively enhance our understanding of the immune response dynamics in COVID-19 and highlight the importance of tailored therapeutic strategies that consider individual variations in cytokine responses, T-cell activation, and B-cell responses. Integrating immunomics into these profiling efforts is crucial because it provides a more detailed and comprehensive analysis, enabling the identification of novel biomarkers and therapeutic targets. Immunomics offers the tools to dissect these complex immune responses at a molecular level, offering insights that are essential for developing effective treatments and preventive measures. Further research using the approaches is necessary to fully elucidate these mechanisms and their implications for treatment and prevention strategies.

### 2.2 Technological advances in immune profiling

Recent technological advancements have substantially improved our ability to detail the immune responses in COVID-19, elucidating the complex dynamics of how the immune system interacts with SARS-CoV-2. Techniques like flow cytometry, mass cytometry (CyTOF), and single-cell RNA sequencing (scRNA-seq) have been central to these discoveries, providing critical data on immune cell activation, cytokine profiles, and genetic markers linked to disease progression (13, 29, 30). These methods are essential components of immunomics, which applies high-throughput technologies to analyze and understand the immune system at a systemic level. CyTOF builds upon flow cytometry by using metal-labeled antibodies to measure over 40 markers on a single cell, providing a detailed assessment of immune cell types in whole blood (31). This technique enables the precise identification of eosinophils, neutrophils, B cells (including plasmablasts and non-plasmablasts), T cells, natural killer (NK) cells, monocytes, dendritic cells (DCs), innate lymphoid cells (ILCs), and immature granulocytes. Neutrophils are distinguished using Cluster of Differentiation (CD) markers such as CD4, CD8, CD14, and CD19, while T cells are characterized by CD14 and CD15. B cells are identified through CD3, CD14, CD15, and CD56. CD3-CD19- cells are further classified by CD3 and CD15. Among T cells, CD8+ T cell subsets are defined by CD27, CD45RA, and CCR7, and similar markers are used to categorize CD4+ T cell subsets. Regulatory CD4+ T cells are marked as CD127low CD25+, whereas follicular CD4+ T cells are identified by CXCR5+ PD-1+. Activation markers are assessed within these memory and non-naïve subsets. Monocytes are classified into classical (CD14++ CD16-), intermediate (CD14+ CD16+), and non-classical (CD14low/- CD16+), while DCs are divided into conventional (CD11c+ CD123low/-) and plasmacytoid (CD11c- CD123+). NK cells are categorized as CD56^bright^ CD16- and CD56^low^ CD16+. By offering a comprehensive analysis of immune cell activation and perturbations, CyTOF enhances our understanding of B cell responses, SARS-CoV-2-specific humoral responses, and T cell activation in relation to disease severity. This advancement allows for the mapping of immune cell populations with unprecedented detail, identifying immune signatures associated with severe COVID-19 cases, often correlating with cytokine storms and adverse clinical outcomes (31). Meanwhile, scRNA-seq offers a granular view of the transcriptomic changes at the single-cell level, revealing the heterogeneity of immune cell responses and pinpointing cell subsets contributing to disease pathology (13). The integration of these technologies into immunomic studies enhances our understanding by linking detailed cellular profiles to clinical outcomes, enabling the identification of novel biomarkers and therapeutic targets. Additional techniques like single-cell Assay for Transposase-Accessible Chromatin using sequencing (scATAC-seq) and multi-omics sequencing approaches have furthered our understanding by exploring epigenetic and molecular changes in immune cells, linking these profiles to patient outcomes and advancing our comprehension of the molecular mechanisms driving disease severity in COVID-19. Li et al. (32) performed single-cell assay for transposase-accessible chromatin using sequencing (scATAC-seq) and single-cell RNA sequencing (scRNA-seq) on peripheral blood mononuclear cells (PBMCs) from severely ill/critical patients (SCPs) with COVID-19, moderate patients (MPs), and healthy controls (HCs). Analyzing 76,570 cells with scATAC-seq and 107,862 cells with scRNA-seq, they identified 28,535 chromatin peaks, with 41.6% in promoter and 10.7% in enhancer regions. Compared to HCs, SCPs and MPs had increased inflammatory pathways, such as the mitogen-activated protein kinase (MAPK) and tumor necrosis factor (TNF) signaling pathways, in CD4+ and CD8+ T cells. SCPs showed reduced accessibility of T-box transcription factor 21 (TBX21) motifs in CD4+ T cells. The scRNA-seq data revealed reduced T cell proportions, especially CD4+ T cells, in SCPs and MPs, with increased expression of inflammatory genes, including nuclear factor kappa B inhibitor alpha (NFKBIA), S100 calcium-binding protein A9 (S100A9), and phosphoinositide-3-kinase regulatory subunit 1 (PIK3R1). CD8+ T cells in SCPs also had upregulated activation markers, such as CD69 and major histocompatibility complex class II (HLA-DRA, HLA-DRB1, HLA-DRB5). Integrated analysis of scATAC-seq and scRNA-seq data showed some consistency. Unterman et al. (14) used single-cell multi-omics, including proteomics and transcriptomics, to examine immune responses in progressive COVID-19. They identified S100Ahi/HLA-DRlo classical monocytes and activated LAG-3hi T cells as markers of disease progression, highlighting abnormal interactions between major histocompatibility complex class II (MHC-II) and lymphocyte-activation gene 3 (LAG-3) on myeloid and T cells. Case studies using these technologies offer practical insights into therapeutic strategies. For instance, Wilk et al. (33) used flow cytometry to identify reconfiguration of peripheral immune cell phenotype in COVID-19, including a heterogeneous interferon-stimulated gene signature, HLA class II downregulation and a developing neutrophil population that appears closely related to plasmablasts appearing in patients with acute respiratory failure requiring mechanical ventilation, and find that peripheral monocytes and lymphocytes do not express substantial amounts of pro-inflammatory cytokines, and then revealed profound T cell depletion in severe COVID-19, emphasizing the need for interventions to enhance T cell function in severely affected patients. Kuri-Cervantes et al. (31) employed CyTOF to explore modulation of the B cell repertoire that its associations with the establishment of a SARS-CoV-2-specific humoral response, and activation of T cells relative to disease severity, and uncover distinct immunological signatures in severe cases, identifying eosinophils, neutrophils, B cells (plasmablasts and non-plasmablasts), T cells, NK cells, monocytes, dendritic cells (DCs), innate lymphoid cells (ILCs) and immature granulocytes in whole blood, and highlighting the expansion of immature neutrophils and inflammatory macrophages, thus providing biomarkers for early detection of severe outcomes (31). Moreover, Liao et al. (13) used scRNA-seq to analyze bronchoalveolar lavage fluid (BALF), identifying 31 distinct cell clusters, including macrophages (CD68), neutrophils (Fc gamma receptor IIIb, FCGR3B), myeloid dendritic cells (mDCs) (CD1C, C-type lectin domain family 9 member A, CLEC9A), plasmacytoid dendritic cells (pDCs) (Leukocyte Immunoglobulin Like Receptor A4, LILRA4), natural killer (NK) cells (Killer Cell Lectin-like Receptor D1, KLRD1), T cells (CD3D), B cells (Membrane Spanning 4-domains A1, MS4A1), plasma cells (Immunoglobulin Heavy Constant Gamma 4, IGHG4), and epithelial cells (Tubulin Polymerization Promoting Protein 3, TPPP3; Keratin 18, KRT18). Major cell types such as mDCs, pDCs, NK cells, T cells, and B cells were present in most samples, with macrophages showing specific enrichment in different groups. BALF from patients with severe or critical COVID-19 had higher proportions of macrophages and neutrophils and lower proportions of mDCs, pDCs, and T cells compared to those with moderate infection. This technology has been crucial in revealing immune cell dysregulation in BALF across different severities of COVID-19, and in identifying potential targets for reducing lung inflammation. These advanced technologies not only provide unprecedented insights into the immune response to SARS-CoV-2, but also highlight specific cellular and molecular targets for potential therapeutic interventions and vaccine development.

### 2.3 Immunological aspects related to biomarkers in COVID-19

Prognostic biomarkers provide valuable insights into the potential progression of COVID-19 to severe conditions like acute respiratory distress syndrome (ARDS) or multi-organ failure. Elevated levels of inflammatory markers such as C-reactive protein (CRP), lactate dehydrogenase (LDH), IL-6, and D-dimer (D-D) are indicative of disease severity and are associated with an increased risk of complications, guiding clinicians in making timely and targeted interventions (24, 34, 35). Genetic markers, particularly those related to the HLA system and ACE2 gene SNPs, offer valuable information regarding susceptibility to severe COVID-19 outcomes (36, 37). Furthermore, research by Wu et al. indicates that SARS-CoV-2 infection leads to lymphocyte suppression and myeloid activation in severe cases, suggesting distinct roles for these cell types in disease progression that should be specifically targeted in treatment strategies (38). Therapeutic biomarkers are crucial for assessing treatment efficacy, allowing for tailored therapies based on changes in cytokine levels or cellular dynamics. This personalized approach maximizes treatment efficacy while minimizing side effects, ultimately optimizing patient care (39). Predictive biomarkers are crucial for forecasting disease progression, allowing doctors to intervene early and tailor treatment strategies. Single-cell analysis has revealed specific markers indicating disease progression, underscoring the importance of precise and personalized medical interventions (14). However, the identification and utilization of biomarkers in managing COVID-19 encounter significant challenges, including variability among patient populations due to factors like age, genetics, and underlying health conditions (25).

### 2.4 Immunomics-driven innovations in COVID-19 vaccine development

Various platforms, including viral vectors, nucleic acids, recombinant proteins with adjuvants, and inactivated viruses, have been leveraged in COVID-19 vaccine development. These innovations have yielded effective vaccines that induce robust immune responses while avoiding the acute inflammation associated with natural SARS-CoV-2 infection (15). Immunomics has played a pivotal role in driving advancements in vaccine technology during the COVID-19 pandemic. Notably, mRNA vaccines instruct cells to produce the SARS-CoV-2 spike protein, eliciting robust immune responses. This approach, informed by immunomic insights, accelerates vaccine design and enhances production scalability (40). Additionally, viral vector vaccines and protein subunit vaccines, informed by immunomic analyses, rapidly induce strong immune reactions (41, 42). Inactivated vaccines, supported by comprehensive immunomic research, ensure safety and efficacy across diverse populations (43). Nanotechnology has further improved mRNA vaccine efficacy by facilitating cellular uptake (44). Integrating immunomics into vaccine development enables precise alignment with global immunological landscapes, ensuring dynamic vaccine customization. Understanding immune memory and implementing booster vaccinations are critical in combating the COVID-19 pandemic. Immunomics, integrating immunological data with omics technologies, provides profound insights into immune memory. Both natural infection and vaccination foster memory B and T cells essential for long-term immunity (45). Booster vaccinations, guided by immunomic analyses, aim to bolster memory responses against waning immunity and emerging variants (46). Immunomics also monitors post-vaccination immune responses, informing vaccine adjustments to enhance effectiveness against evolving strains (31). This adaptability is crucial for maintaining vaccination program efficacy amidst viral mutations. Robust genomic surveillance facilitated by immunomics is essential for tracking SARS-CoV-2 variants and understanding immune evasion mechanisms. Harvey et al. and McLean et al. reviewed how advanced immunomics techniques map the altered immune landscapes induced by variants (41, 47). These techniques reveal antigenic drift and conformational changes in spike proteins, which contribute to immune escape (47). Furthermore, mutations in T-cell epitopes allow variants to evade cellular immune responses, highlighting the virus’s adaptive nature (48). Global genomic surveillance, informed by immunomics, enables real-time adaptation of vaccine strategies and public health measures to counteract the challenges posed by viral mutations (49). Through immunomics, researchers maintain a dynamic response to the pandemic, ensuring effective vaccine strategies and public health interventions.

### 2.5 Identification of immunotherapeutic targets

Immunomics plays a pivotal role in identifying novel immunotherapeutic targets crucial for managing infectious diseases like COVID-19. By unraveling intricate pathogen-host immune system interactions, immunomics lays the groundwork for tailored treatment strategies, significantly enhancing patient outcomes (50). One critical target for immunotherapeutic interventions is the interaction between the SARS-CoV-2 spike protein and the ACE2 receptor on human cells, which has been extensively studied to block or modulate this interaction (51). Additionally, targeting the immune response, particularly in managing the cytokine storm syndrome observed in severe COVID-19 cases, is a significant area of research (24). Clustered regularly interspaced short palindromic repeats (CRISPR) screens and proteomics have been instrumental in identifying potential immunotherapeutic targets, accelerating target discovery (52). Computational models and systems biology aid in predicting therapeutic targets and understanding their mechanisms of action, integrating vast datasets from immunomic studies (53). From an immunomics perspective, monoclonal antibodies (mAbs) represent a breakthrough in COVID-19 treatment strategies. Engineered to target the SARS-CoV-2 virus, mAbs mimic the body’s natural immune defenses by focusing on critical viral antigens, primarily the spike protein (54). Clinical trials leveraging immunomic techniques have demonstrated the robust efficacy of mAbs in reducing viral load, alleviating symptoms, and decreasing hospitalization and mortality rates among COVID-19 patients (54). However, challenges such as high production costs, logistical complications, and the emergence of viral variants threaten their efficacy (54). Yet, ongoing integration of immunomic insights is essential for adapting these therapies to emerging variants, ensuring sustained effectiveness (54). Personalized immunotherapy, guided by immunomic data, enhances therapy efficacy and reduces side effects. Biomarkers from patients inform the selection and dosage of therapeutic agents, enabling tailored treatment strategies (55). Personalized therapies, including mAbs targeting specific antigens unique to individual infections and cytokine inhibitors tailored to patient cytokine profiles, offer a customized approach to managing the immune response (56). COVID-19 convalescent plasma (CCP) has also shown promise as a safe and potentially effective therapeutic option for high-risk outpatients (57). The deployment of personalized immunotherapies faces logistical hurdles, including complex and costly manufacturing processes and ethical considerations regarding equitable access (58). Current technologies face challenges in accurately and efficiently identifying therapeutic targets and integrating vast amounts of data to inform treatment strategies (59). Overcoming these challenges requires continued advancements in technologies to ensure the widespread accessibility and effectiveness of personalized immunotherapies.

## 3 Immunomic analysis of animal hosts in SARS-CoV-2 transmission

### 3.1 Role of animals in SARS-CoV-2 epidemiology

In the epidemiology of SARS-CoV-2, animals serve critical roles as both reservoirs and vectors (8, 60–63). Bats are considered primary reservoirs for SARS-CoV-2 due to their extensive viral diversity and unique immune features that allow them to harbor viruses without becoming ill (8, 62). Genetic analyses strongly suggest bats as natural reservoirs, given the substantial genetic similarities between bat coronavirus RaTG13 and SARS-CoV-2 (8). Additionally, similar coronaviruses have been identified in pangolins, supporting the hypothesis that these mammals could be intermediate hosts facilitating transmission to humans (60). Vectors, on the other hand, are organisms that do not necessarily support the long-term survival of a virus within their bodies but can transmit the virus between susceptible hosts (63). Minks, for example, have been implicated in transmitting the virus to humans and other minks, suggesting their role as both reservoirs and vectors (7, 63, 64). This dual function facilitates broader dissemination of the virus. Recent studies have highlighted SARS-CoV-2 infections in various domestic and wild animals, revealing complex epidemiological dynamics. Significant outbreaks in mink farms underscore the need for robust animal surveillance to monitor potential reservoirs that could contribute to new infection chains in humans (7). The persistent presence of SARS-CoV-2 in many animal species presents ongoing risks for zoonotic transmission and reverse zoonosis, where humans infect animals, potentially establishing new viral reservoirs and complicating control measures (62). An integrated One Health approach is paramount for managing these zoonotic risks, integrating veterinary health, wildlife management, and public health initiatives to develop coordinated surveillance and response mechanisms (58).

### 3.2 Immunomic techniques revealing cross-species transmission mechanisms

Advanced immunomic techniques are essential for elucidating how animals function as reservoirs and vectors for SARS-CoV-2 and for uncovering the specific mechanisms of its transmission. These techniques facilitate the detection of antibodies indicative of past infections and enable genomic sequencing to track changes in the viral genome (65). By integrating high-throughput immunological assays and omics technologies, researchers can dissect immune responses across species and identify adaptive changes that enable the virus to infect new hosts. Recent metagenomic sequencing of frozen tissue samples from pangolins has highlighted significant sequence similarities between human SARS-CoV-2 and coronaviruses in pangolins and bats (60). This research underscores the importance of ACE2 receptor variations across species, which influence the virus’s ability to bind and enter host cells, affecting its host range (66). Immunomic studies have revealed how these receptor variations impact viral cross-species transmission. Understanding SARS-CoV-2 transmission involves distinguishing between major avenues and specific mechanisms. Major transmission avenues include direct interactions and indirect influences on transmission dynamics. Direct interactions involve animal-to-human, animal-to-animal, and human-to-animal transmission through physical contact, such as handling infected animals or consuming contaminated animal products. Significant outbreaks in mink farms illustrate this direct transmission route, with studies using flow cytometry to reveal immune cell activation patterns in infected minks (7). Indirect influences include environmental factors like fomites, behavioral practices, and social interactions. SARS-CoV-2 has been detected on surfaces, where it can survive and infect new hosts through contact with contaminated surfaces (67). High-throughput serological testing has detected antibodies in various animals, indicating past infections and potential ongoing transmission (65). These influences contribute to the four major transmission modes: direct physical contact, indirect contact (fomites), large droplets, and fine aerosols.

In addition, the variability in immune responses, such as cytokine release patterns and immune cell activation, is crucial for understanding the virus’s adaptation to new hosts. Research indicates that cats, ferrets, and some deer are highly susceptible and may act as virus carriers, while dogs exhibit lower susceptibility and reduced transmission capabilities (62). Advanced immunomic techniques, including scRNA-seq, have been employed to map immune responses in animals, providing insights into cellular-level interactions with the virus (68). Integrating these findings within the One Health framework has greatly improved our understanding of SARS-CoV-2 transmission (64, 69). This approach emphasizes the importance of monitoring various animal species to understand their role in the virus’s ecology and transmission chains. Combining genetic and immunomic studies enhances our understanding of zoonotic transmission pathways and informs public health strategies and preventive measures.

### 3.3 Studies on reservoirs, intermediate hosts, and animal immune responses

Research integrating genetic and immunomic findings is crucial for identifying potential reservoirs and intermediate hosts, which is essential for predicting and mitigating risks associated with emerging infectious diseases. For example, significant outbreaks in mink farms have highlighted the virus’s ability to infect these animals and mutate, posing a potential public health risk through possible transmission back to humans (68). Advanced immunomic techniques, combined with genomics and bioinformatics, are pivotal for monitoring viral presence and evolution in animal populations. These approaches aid in mapping the spread of SARS-CoV-2 and understanding species-specific immune responses (70). Studies on immune responses in animal models have been instrumental in uncovering new therapeutic targets and advancing treatments. Research involving mice engineered to express human ACE2 receptors has provided valuable insights into viral replication and immune responses, which are crucial for guiding vaccine development (71). Additionally, investigating immune responses in wildlife and domestic animals that show resistance to viral infections can reveal unique mechanisms and potential therapeutic targets. For instance, bats, despite being natural reservoirs of SARS-CoV-2, exhibit minimal disease symptoms, suggesting immune mechanisms that could inspire novel treatments (62). Furthermore, animal models, including mice and non-human primates, are essential for studying the virus’s progression and accelerating the development of vaccines and therapeutics. Mice expressing human ACE2 receptors closely mimic human viral entry points, while ferrets offer valuable data on virus transmission and respiratory impacts (72). Non-human primates, such as rhesus macaques, closely replicate human disease, aiding in vaccine and therapeutic evaluations (16). An integrated One Health approach, which acknowledges the interconnectedness of animal, human, and environmental health, is crucial for preventing and controlling the spread of SARS-CoV-2 and achieving optimal health outcomes. Additionally, high-throughput sequencing technologies enhance our ability to map viral mutations in animal hosts, aiding in the prediction and prevention of potential spillover events to humans.

### 3.4 Integrated one health approaches to managing COVID-19 risks and animal protection

An integrated One Health approach is crucial for managing zoonotic risks such as COVID-19, by merging veterinary health, wildlife management, and public health initiatives to create coordinated surveillance and response mechanisms. This approach is vital for understanding and controlling zoonotic diseases, like COVID-19, as wildlife reservoirs can harbor viruses with minimal illness to the hosts, thereby serving as long-term sources of infection. Effective response mechanisms include strategies to manage and mitigate the risk of disease transmission from these reservoirs to humans, which involve monitoring, surveillance, habitat management, and population control measures (73). Population control, aimed at managing specific wild animal populations to reduce human contact, is a delicate issue that requires careful consideration within the One Health framework to balance the health of animals, humans, and ecosystems. This is particularly important for protected species such as bats and pangolins, which have significant cultural and ethical implications (74). Monitoring wild animal populations provides essential data for predicting and preventing outbreaks by identifying potential zoonotic reservoirs and understanding viral transmission dynamics. However, this must be done with minimal disruption to wildlife and their habitats to prevent stressing animal populations and disrupting ecological balances, which could lead to habitat loss or decreased biodiversity. Avoidance strategies are also crucial in reducing human-wildlife contact to prevent virus spillover. These strategies include preserving habitats to reduce human encroachment, educating the public about the risks of wildlife contact, enforcing stricter regulations on wildlife trade, and employing non-invasive monitoring technologies such as remote sensing, camera traps, and environmental DNA sampling (75). This holistic approach addresses the interconnected health of humans, animals, and ecosystems, enhancing our ability to control the spread of SARS-CoV-2, mitigate future outbreaks, and bolster global health security.

## 4 Environmental factors and immunomics in SARS-CoV-2 spread

### 4.1 The impact of air quality on SARS-CoV-2 transmission and immune response

Air quality profoundly influences the transmission dynamics of respiratory viruses such as SARS-CoV-2. Poor air quality, characterized by elevated levels of particulate matter (PM_2.5_ and PM_10_), can enhance viral transmission by acting as a carrier for viral particles, thereby extending their range and increasing the spatial distribution of COVID-19 cases in urban environments (76, 77). Fine particulate matter, in particular, adsorbs viral particles, potentially enhancing their longevity and infectiousness. SARS-CoV-2 viral RNA has been detected in fine aerosols (<5 microns), with a half-life of about one hour, suggesting that these airborne particles can remain infectious (78). Evidence of SARS-CoV-2 particles on surfaces like air exhaust outlets and fans further supports airborne transmission in settings where direct contact with an infected individual is unlikely (79). Poor air quality, characterized by high levels of particulate matter (PM_2.5_) and harmful gases, can compromise mucosal immunity and heighten vulnerability to respiratory infections like COVID-19 (76). Fine particulate matter can also adsorb viral particles, potentially enhancing the virus’s stability and spread (77). Studies have shown that improved ventilation and air cleaning significantly reduce airborne viral loads, thereby mitigating the risk of transmission within enclosed spaces (80, 81). Effective ventilation, whether through natural means like opening windows or mechanical systems, helps disperse and dilute viral particles, reducing their potential to infect new hosts.

Beyond its role in viral transmission, air quality also critically impacts immune health. Pollutants such as particulate matter and harmful gases can impair mucosal immunity, making individuals more susceptible to respiratory infections like COVID-19. Chronic exposure to air pollutants exacerbates inflammatory responses and weakens the immune system, leading to more severe disease outcomes (10, 11, 76, 77). Compromised immunity can result in heightened susceptibility to infections and poorer disease prognosis. During the COVID-19 pandemic, research has highlighted that managing indoor air quality is crucial not only for controlling viral transmission but also for protecting immune health. Enhanced ventilation and air purification are essential strategies for reducing airborne viral particle concentrations and improving overall public health safety. Addressing both the environmental and immune factors involved in SARS-CoV-2 transmission underscores the importance of comprehensive public health strategies that integrate air quality management with immune health considerations to effectively combat the spread of COVID-19.

### 4.2 The impact of water systems on SARS-CoV-2 transmission and immune response

The stability and transmission dynamics of SARS-CoV-2 are significantly influenced by water systems and their interactions with immune responses. Research has demonstrated that SARS-CoV-2 can be detected in untreated wastewater, indicating that sewage systems may act as reservoirs and potentially contribute to transmission if not adequately managed (82). Wastewater-based epidemiological surveillance has become an essential tool for estimating community infection prevalence and providing early warnings for outbreaks (83). Although direct waterborne transmission is limited, the presence of SARS-CoV-2 RNA in sewage underscores the importance of effective sanitation and water treatment practices to mitigate potential risks. Disinfection methods, such as chlorination, have been shown to effectively inactivate the virus in water, thereby reducing transmission risks. Furthermore, water systems can impact immune responses indirectly. The quality of drinking water and the effectiveness of water treatment play critical roles in influencing overall health and immune function. For instance, hydrogen-rich water has been reported to reduce inflammatory responses and prevent apoptosis of peripheral blood cells in healthy adults (84). Poor water quality can contribute to the burden of infections and illnesses, which can undermine immune responses and increase susceptibility to severe outcomes of SARS-CoV-2 infection. Microbial contamination from community water systems, such as waterborne Legionella and non-tuberculous mycobacteria, has been shown to significantly impact human health (85). These contaminants can exacerbate the health burden, potentially compromising immune function and making individuals more vulnerable to severe infections, including those caused by SARS-CoV-2 virus more susceptible to severe outcomes of SARS-CoV-2 infection. Maintaining effective sanitation and water treatment is crucial for minimizing the risks associated with waterborne pathogens and supporting public health. Implementing robust water management strategies and enhancing wastewater surveillance can play a vital role in controlling SARS-CoV-2 transmission and safeguarding community health.

### 4.3 The impact of other environmental factors on SARS-CoV-2 transmission and immune response

In addition to air quality and water systems, various other environmental factors significantly influence SARS-CoV-2 transmission and immune responses. Temperature plays a key role; lower temperatures enhance the virus’s stability, allowing it to persist longer on surfaces and in the air, which can increase transmission rates during colder months (86, 87). Conversely, higher temperatures accelerate viral degradation, reducing its viability. Humidity levels also affect the stability of the virus. Low humidity can prolong the presence of SARS-CoV-2 in the air, whereas higher humidity helps respiratory droplets settle more quickly, thereby reducing airborne transmission (86, 87). The type of surface material also influences the persistence of SARS-CoV-2, with the virus surviving longer on materials like plastic and stainless steel compared to copper and cardboard. This understanding is crucial for developing effective cleaning and disinfection strategies (67). UV light, particularly UV-C, has been shown to effectively inactivate SARS-CoV-2, demonstrating the benefits of natural sunlight and UV-C disinfection in reducing viral viability (88). Environmental stressors, such as noise and overcrowding, also compromise immune function, altering susceptibility to and progression of viral infections like SARS-CoV-2 (89). Chronic exposure to these stressors can impair immune function, increasing susceptibility to severe disease outcomes (89). Understanding the interactions between environmental factors and immune responses is essential for developing effective public health interventions. Managing environmental factors and improving air quality can help reduce SARS-CoV-2 transmission and enhance overall public health safety. Public health guidelines should incorporate these insights to effectively manage environmental conditions and mitigate the spread of the virus.

### 4.4 High-throughput immunomic technologies in environmental health

High-throughput immunomic profiling technologies, such as CyTOF and single-cell RNA sequencing, are essential for detailing immune responses under diverse environmental conditions, particularly amidst the COVID-19 pandemic. These technologies provide precise immune cell characterization, offering insights into how air quality and climate change affect immune profiles and responses to SARS-CoV-2 (90). These advanced technologies enable comprehensive analysis of environmental samples to detect the presence of SARS-CoV-2 and other pathogens. They also help understand how environmental exposures affect immune system functioning. For example, high-throughput sequencing could identify microbial communities and their interactions with the virus in various environments, providing insights into potential hotspots for transmission.Karczewski et al. (91) compared two methods for analyzing microbial diversity in groundwater ecosystems: Terminal Restriction Fragment Length Polymorphism (T-RFLP) and high-throughput sequencing (HTS). Their study assessed the effectiveness of these techniques in evaluating microbial community composition and diversity. Fontenele et al. (92) utilized high-throughput sequencing to analyze SARS-CoV-2 in wastewater. They employed Illumina MiSeq and NextSeq platforms to sequence viral RNA, providing detailed insights into circulating variants. This approach demonstrated the potential of wastewater-based surveillance for monitoring SARS-CoV-2 prevalence and mutations in communities, highlighting how HTS can enhance understanding of virus dynamics. ScRNA-seq allows for the detailed examination of immune cell responses at the individual cell level, revealing how environmental factors such as pollutants impact immune functionality. CyTOF, or mass cytometry, combines flow cytometry and mass spectrometry, allowing for the simultaneous measurement of multiple parameters on individual cells. Bioinformatics synthesizes environmental data with immunological outcomes, essential for predicting disease spread and severity under varied environmental conditions. Advanced algorithms and machine learning techniques analyze large datasets, identifying patterns and correlations that can inform public health strategies. Integrating immunomic data with environmental monitoring helps develop predictive models for outbreaks, refine public health interventions, and enhance environmental controls.

### 4.5 Integrated immunological and environmental factors approaches to managing COVID-19 risks

Environmental factors play a significant role in the transmission dynamics of SARS-CoV-2. Elements such as population density, urbanization, environmental variables, and environmental pollution can enhance the spread of the virus. Crowded urban areas with high population densities facilitate close contact among individuals, increasing transmission rates. Additionally, environmental pollution, including particulate matter and other pollutants, may affect respiratory health and increase susceptibility to viral infections (10). Research conducted in various urban areas during the pandemic indicated a correlation between high pollution levels and increased COVID-19 case numbers. Research has established that meteorological variables such as temperature, humidity, and air quality significantly influence immune responses to SARS-CoV-2, thereby affecting viral transmission rates and immune defenses. Davis et al. (93) emphasize that immunomics enhances the understanding of dynamic immune interactions, which is essential for developing interventions for infectious diseases like COVID-19. Mecenas et al. (11) highlights how these factors impact infection severity and distribution. Integrating environmental and immunological data enables better anticipation and mitigation of environmental impacts on COVID-19 spread and severity. Such insights refine public health interventions and enhance predictive models for outbreaks.

By synthesizing environmental data with epidemiological insights, our review offers insights on how natural and human-modified environments affect the dynamics of SARS-CoV-2 transmission. For instance, Naidoo et al. reported that overlaying virus stability data with weather patterns and human mobility data helps predict potential hotspots and inform public health responses (94). Immunology, employing advanced techniques, intricately dissects the immune system’s complexities, particularly its interplay with environmental factors. This field unravels the intricate networks of immune signaling and responses at the molecular level, providing profound insights into how external conditions shape immune functionality. These specific examples highlight the critical role of environmental factors in understanding and controlling the spread of COVID-19. Integrating environmental and immunological data enables better anticipation and mitigation of environmental impacts on COVID-19 spread and severity. Such insights refine public health interventions and enhance predictive models for outbreaks. This holistic approach not only informs immediate response strategies but also aids in preparing for future outbreaks by emphasizing the integration of environmental health in pandemic preparedness.

## 5 Integrating immunomics within the one health framework for COVID-19

### 5.1 Overview of immunomics and one health integration

The integration of immunomics within the One Health framework represents a comprehensive approach to managing pandemics like COVID-19, emphasizing the interconnected health of humans, animals, and the environment (12). This interdisciplinary model enhances our understanding of zoonotic diseases and strengthens global health security by promoting data and resource sharing across borders (6). It encourages multidisciplinary collaboration, essential for devising strategies to predict, prevent, and manage infectious diseases effectively (95). Immunomics, as a crucial component of this framework, provides in-depth insights into the immune responses of various species to SARS-CoV-2, helping identify potential reservoirs and understand transmission dynamics (9, 64). This information is vital for developing targeted strategies to control disease spread and integrating environmental data to better understand how ecological factors influence transmission. By incorporating these insights, the One Health approach facilitates the development of robust public health policies and enhances preparedness for future outbreaks. It builds resilience against zoonotic challenges and improves health security measures, necessitating a coordinated strategy that includes surveillance, data analytics, and ecological considerations (96).

### 5.2 Case studies of practical applications

The integration of the One Health framework with immunomics has significantly enhanced our understanding of the COVID-19 pandemic, enabling targeted interventions based on high-throughput immunomics data. This approach provides comprehensive insights into the disease’s complexities and informs practical response strategies. For instance, research by Zhou P. et al. (53) employed NGS to identify the genetic sequence of SARS-CoV-2 from clinical samples collected from patients with pneumonia. They conducted phylogenetic analyses comparing these sequences with those of other coronaviruses. The study revealed a coronavirus in bats that is genetically similar to SARS-CoV-2, suggesting that bats are a natural reservoir. This finding has been instrumental in tracing the zoonotic origins of the virus and underscores the importance of wildlife monitoring to prevent future outbreaks. Complementary immunomics analysis such as high-throughput sequencing to analyze viral RNA sequences analysis extended to pangolins, as documented by Lam TT et al. (60), revealing that pangolins host coronaviruses closely related to SARS-CoV-2, shedding light on potential intermediate hosts and transmission dynamics. Furthermore, the intersection of immunomics and One Health has been essential in examining environmental factors influencing viral transmission and immune responses. Research by Ciencewicki J. and Jaspers I (10). demonstrated how air pollution can compromise immune responses in the respiratory tract, increasing susceptibility to respiratory virus infections and potentially exacerbating disease severity. Their study utilized cytokine profiling and immune cell analysis to show how pollutants affect inflammatory pathways and immune function. Research by Mecenas P. et al. (11) investigated the impact of climate change on the stability and transmission rates of viruses like SARS-CoV-2.They emphasized the importance of integrating immunomic data with environmental factors to predict the risk levels for different regions and populations under various climate scenarios. Their research involved correlating viral load and immune response data with temperature and humidity variations to assess their effects on viral spread. The integration of immunomics within the One Health framework has also deepened our understanding of animal reservoirs and their roles in virus spillover to humans. A study by Andersen KG et al. (70) explored the genomic characteristics of SARS-CoV-2 in relation to other coronaviruses found across different species. This research, augmented by proteomic profiling and structural analysis, has enabled scientists to identify specific viral mutations that facilitate cross-species transmission, providing insights into how SARS-CoV-2 adapts to new hosts. Recent studies have underscored the significant role of agricultural settings and wildlife interfaces in the transmission dynamics of zoonotic diseases, including COVID-19. A study by Oreshkova N. et al. (7) using genetic sequencing of SARS-CoV-2 strains analysis investigated SARS-CoV-2 infections in mink farms in the Netherlands, documenting transmission from humans to minks and identifying potential mutations that could affect the virus’s transmissibility and virulence. The research demonstrated that SARS-CoV-2 spread rapidly among minks and was transmitted back to humans, highlighting the potential role of animal farms as reservoirs for the virus. By applying immunomic techniques to analyze immune responses in minks, researchers can identify specific immunological markers that indicate spillover potential. These case studies illustrate how integrating immunomics with the One Health approach facilitates proactive prevention and mitigation of pandemic impacts through strategies that are both informed and data-driven, rather than merely reactionary.

### 5.3 Future research directions and public health strategy implications

To effectively integrate immunomics and the One Health framework in addressing COVID-19 and similar zoonotic diseases, we posit that future research and technological advancements should achieve significant breakthroughs in the following areas. (1) Develop cost-effective immunomic technologies. Future research must prioritize the development of cost-effective, scalable, high-throughput immunomic technologies. Transformative advancements like single-cell RNA sequencing and mass cytometry face limitations such as high costs and operational complexity, hindering widespread use in resource-limited settings. Complex data from these technologies require sophisticated computational tools for analysis, posing challenges in distinguishing meaningful signals from noise. The specificity and sensitivity of biomarkers also limit their clinical utility, risking disease misclassification and missed early detection, crucial for effective interventions. Moreover, scalability remains an issue, especially during pandemics, where rapid application to large populations is constrained by high costs and logistical complexities. Addressing these challenges necessitates a holistic approach that integrates technological innovation, enhances data analysis capabilities, and improves accessibility across global healthcare systems. (2) Enhance data analysis capabilities. Leveraging artificial intelligence (AI) and machine learning (ML) can significantly refine our ability to interpret complex immune response data, enabling the identification of novel biomarkers and optimizing the analysis of genetic, proteomic, and metabolic information to predict individual treatment outcomes and disease progression (10). Integrating sophisticated computational tools with clinical research will facilitate the rapid identification and validation of therapeutic targets in personalized medicine. (3) Expand immunomic databases. Broadening immunomic databases to encompass a wider array of demographic variables will improve the tailoring of treatments to individual genetic and immunological profiles, enhancing treatment efficacy and minimizing adverse effects. (4) Integrate Environmental and Immunomic Data. In the context of One Health, immunomics will advance our understanding of how environmental factors influence zoonotic disease transmission. Innovations such as spatial transcriptomics and advanced ML models will transform our ability to predict interactions between pathogens and their hosts within environmental contexts, providing critical insights that could preempt future outbreaks (8). However, isolating the effects of specific environmental factors on immune responses is challenging due to the intricate nature of environmental-immunological interactions. Understanding these interactions demands sophisticated experimental and analytical methodologies to establish clear causal relationships. The complexity of these interactions often obscures the direct impacts of environmental changes on disease dynamics and immune system behavior (11). In summary, integrating large-scale environmental and immunomics datasets introduces substantial challenges. The vast volume and diversity of the data necessitate advanced analytical techniques to decipher meaningful patterns and derive actionable insights. This process is essential for translating complex data sets into practical applications in public health and epidemiology. Ultimately, integrating immunomics data within a One Health framework enhances the effectiveness of global response strategies and pandemic preparedness.

## 6 Methodological strategies for data collection

In this study, we analyzed 61 original research papers and 36 review articles. The original research papers include 23 studies related to immunomics, 16 papers focused on immunology, 1 paper addressing One Health, and 21 other relevant studies. Our analysis included a diverse array of biological samples, such as peripheral blood mononuclear cells (PBMCs), whole blood (WB) samples, BALF immune cells, lung specimens, serum, nasal secretions, feces, saliva, urine, and swabs from various sites (nose, throat, oral, rectal), as well as cell lines. The comprehensive sampling across these studies ensures sample integrity and representativeness, which are for our study. These details are provided in **Supplementary Table 1**.

Significantly, we examined major types of data including genomics (7, 8, 36, 49, 60, 70), transcriptomics (13, 14, 21, 33, 38), proteomics (14, 15), and bioinformatics (7, 8, 14, 37, 46, 49, 51, 60, 65, 66, 70, 97). We also reviewed key associated techniques such as NGS, CyTOF, and scRNA-seq, RT-PCR, and ELISA, which have been pivotal in understanding the molecular mechanisms of immune responses. These methods provide essential data on immune cell activation, cytokine profiles, and genetic markers linked to disease progression. These studies in our review, including those using NGS (7, 8, 36), have played a crucial role in identifying genetic variations that dictate individual responses to diseases and vaccines. Flow cytometry, used traditionally to analyze the physical and chemical characteristics of cells, was highlighted in studies like those by Wilk et al. (33) Additionally, CyTOF, which builds upon flow cytometry by using metal-labeled antibodies to measure over 40 markers on a single cell, has provided insights into distinct immunological signatures in severe cases, as shown by Kuri-Cervantes et al. (31). ScRNA-seq offers a detailed view of transcriptomic changes at the single-cell level, revealing the heterogeneity of immune cell responses and pinpointing cell subsets contributing to disease pathology. This technique, along with scATAC-seq and multi-omic sequencing approaches, has advanced our understanding by exploring epigenetic and molecular changes in immune cells, linking these profiles to patient outcomes and furthering our comprehension of the molecular mechanisms driving disease severity in COVID-19 (14, 32). Bioinformatics synthesizes these diverse datasets, enabling complex analyses that forecast disease progression, tailor treatments, and facilitate the development of targeted vaccines and immunotherapies (8, 14, 37, 46, 49, 51, 60, 65, 66, 70, 97). This field also reviews technological tools such as RT-PCR and ELISA, analyzing cytokine trends for identification and treatment of hyperinflammation, displaying the immune responses of the rSARS-CoV-2 vaccine, and exploring SARS-CoV-2 transmission models (24, 42, 64).

Data resources are detailed in **Supplementary Table 1**. For instance, the dataset from the study by Zhao et al. (21) is accessible available on CNGB Nucleotide Sequence Archive (CNSA) with accession number CNP0001250. The datasets from Penttilä PA et al.’s study (19) are available in the Flow repository and on GitHub (https://flowrepository.org/id/FR-FCM-Z34U and https://github.com/saeyslab/CYTOF_covid19_study). All original research received the necessary ethics approval and adhered to appropriate guidelines. To ensure data accuracy and reliability, both raw and processed data are available in the corresponding repositories, subject to management regulations. These datasets can be accessed for further analysis in compliance with data protection regulations.

## 7 Conclusion

This review integrates the application of immunomics within the One Health framework, highlighting its critical role in addressing the multifaceted challenges posed by the COVID-19 pandemic. Our analysis demonstrates the profound utility of immunomics in dissecting complex immune responses across species, including humans and animals, and the significant environmental factors influencing viral propagation and disease manifestation. Immunomic analyses have provided invaluable insights into distinct immune mechanisms triggered by COVID-19 in humans, identifying key biomarkers indicative of disease severity and susceptibility. These findings are instrumental in refining clinical interventions and enhancing therapeutic outcomes. Additionally, we have explored the roles of various animal species as reservoirs or vectors in the virus’s transmission chain, emphasizing the necessity of stringent wildlife monitoring and ecosystem management to curtail zoonotic spillovers. Furthermore, we have examined how environmental variables, such as air quality and water systems, impact viral stability and dissemination, advocating for stringent environmental monitoring and proactive public health interventions to mitigate these influences. The review also discusses the transformative impact of advanced omics technologies on accelerating vaccine development and enhancing the detection and monitoring of viral mutations. These technological advancements are pivotal for navigating the virus’s evolutionary trajectory and its potential for cross-species transmission. In summary, the synthesis of immunomics within the One Health framework emerges as a transformative strategy for enhancing pandemic preparedness and response. By broadening the scope of immunomic studies to include a more extensive array of biodiversity and ecological settings, we can unlock novel insights into virus-host dynamics and mechanisms of zoonotic spillover, thereby fortifying our global preparedness for emerging health challenges.
