# Supplementary material for: Immunomics in one health: understanding the human, animal, and environmental aspects of COVID-19
Source: Front Immunol. 2024 Sep 4;15:1450380. doi: 10.3389/fimmu.2024.1450380 (PMC11408184; doi:10.3389/fimmu.2024.1450380)
Supplement: Supplementary file 1 [file Table1.docx]

Supplementary Material

# Supplementary Figures and Tables

Supplementary Table 1**:** Full table of overview of immunomics, one health, and related insights from the publications in this review.

| **Authors** | **PMID** | **Publication Date** | **Type** | **Subjects** | **Biospecimen type** | **Immunology** | **Omics** | **High-throughput techniques** | **Other** | **One health** | **Main Results** | **Data Resources** |
| --- | --- | --- | --- | --- | --- | --- | --- | --- | --- | --- | --- | --- |
| Oreshkova N, et al. [7] | 32553059 | 11-Jun-20 | Environmental, Human, Animal | 36 minks with COVID-19 | Lung specimens | - | Genomics, Bioinformatics | Next-generation sequencing | - | Yes | underscore the need for robust animal surveillance to monitor potential reservoirs contributing to new infection chains in humans. | The viral sequences of the index samples and from additional four and five animals from NB1 and NB2, respectively, were determined by next generation sequencing and deposited in GenBank (MT396266 and MT457390-MT457399). |
| Zhou P, et al. [8] | 32015507 | 3-Feb-20 | Human, Animal | 7 patients with COVID-19 | Human samples, including oral swabs, anal swabs, blood and BALF samples, | - | Genomics, Bioinformatics | Next-generation sequencing | - | Yes | Genetic analyses strongly suggest bats as natural reservoirs due to substantial genetic similarities between bat coronavirus RaTG13 and SARS-CoV-2, indicating an evolutionary link. | Sequence data that support the findings of this study have been deposited in GISAID (https://www.gisaid.org/) with accession numbers EPI_ISL_402124, EPI_ISL_402127–EPI_ISL_402130 and EPI_ISL_402131; GenBank with accession numbers MN996527–MN996532; National Genomics Data Center, Beijing Institute of Genomics, Chinese Academy of Sciences (https://bigd.big.ac.cn/databases?lang=en) with accession numbers SAMC133236–SAMC133240 and SAMC133252. |
| Halfmann PJ, et al. [9] | 32402157 | 13-May-20 | Human, Animal | Three cat pairs | Nasal and rectal swabs | - |  | - | - | Yes | provides in-depth insights into the immune responses of various species to SARS-CoV-2, helping identify potential reservoirs and understand transmission dynamics. | DOI: 10.1056/NEJMc2013400. |
| Rüegg SR, et al. [12] | 28261580 | 16-Feb-17 | Environmental, Human, Animal | null | null | - |  | - | - | Yes | The framework identifies the social, economic, and environmental drivers leading to integrated approaches to health and illustrates how these evoke characteristic One Health operations, i.e., thinking, planning, and working, and require supporting infrastructures to allow learning, sharing, and systemic organization. | - |
| Liao M, et al. [13] | 32398875 | 12-May-20 | Human | 13 patients with COVID-19 | Bronchoalveolar lavage fluid (BALF) immune cells | Proinflammatory monocyte-derived macrophages | Transcriptomics | Single-cell RNA sequencing (scRNA-seq) | - | No | provides a deep dive into the atlas of the bronchoalveolar immune microenvironment which uncover the potential mechanisms underlying pathogenesis and recovery in COVID-19. | All data used in this study, including scRNA-seq and scTCR-seq raw data, filtered expression matrix and scTCR-seq contig annotation that support the findings of this study can be accessed in GEO under the accession number GSE145926. |
| Unterman A, et al. [14] | 35064122 | 21-Jan-22 | Human | 10 patients with COVID-19 and 13 healthy subjects | Peripheral blood mononuclear cells (PBMCs) | Immune cells | Proteomics, Transcriptomics, Bioinformatics | Single-cell multi-omics | - | No | probes immune profiling about dyssynchrony of the innate and adaptive immune interaction in progressive COVID-19. | Raw data have been deposited in the GEO database under accession code GSE155224. The results can be further explored through the COVID-19 Cell Atlas Data Mining Site (www.covidcellatlas.com). |
| Ivanova EN, et al. [15] | 38213787 | 23-Nov-23 | Human | 38 acute and 38 convalescent COVID-19 patients | Peripheral blood mononuclear cells (PBMCs) | Cytotoxic gene and immune cells | Proteomics | Single-cell RNA-seq | - | No | provides a deep dive into the cytotoxic gene and immune cells from COVID-19 patients and healthy volunteers receiving the SARS-CoV-2 vaccine and booster, shedding light on how different clonal expansion among effector cells in COVID-19 patients and memory cells in vaccine recipients. | Data can be explored interactively through the web at https://cellxgene.cziscience.com/. |
| Munster VJ, et al. [16] | 32396922 | 12-May-20 | Animal | 8 adult rhesus macaques | Blood, nose swab | Animal model of COVID-19 |  | - | - | No | aiding in vaccine and therapeutic evaluations. | Data have been deposited in Figshare: https://doi.org/10.35092/yhjc.12026910. |
| Lucas C, et al. [17] | 32717743 | 27-Jul-20 | Human | 113 patients with moderate or severe COVID-19 | Peripheral blood mononuclear cells (PBMCs) | cytokines and immune cells |  | - | - | No | provides a deep dive into the inflammatory responses triggered by COVID-19, shedding light on how different cytokines and immune cells contribute to disease severity. It also emphasizes the complexity of immune responses, including type I interferon signaling in the context of COVID-19. | Uploaded in ImmPort (https://www.immport.org/shared/home, Study ID: SDY1655) |
| Mathew D, et al. [18] | 32669297 | 15-Jul-20 | Human | 60 healthy donors (HDs), 36 recovered donors (RDs), and 125 hospitalized COVID-19 patients | Peripheral blood mononuclear cells (PBMCs) | Immune perturbations |  | - | High-dimensional flow cytometry | No | identifys three distinct immunotypes of lymphocyte reactions in hospitalized patients, correlating with disease severity, and illustrates a complex relationship between immune response and COVID-19. | Flow cytometry data collected in this study were deposited to the Human Pancreas Analysis Program (HPAP-RRID:SCR_016202) Database and Cytobank (61) (https://hpap.pmacs.upenn.edu): B cell data (https://premium.cytobank.org/cytobank/experiments/308353), non-naïve CD4 T cells (https://premium.cytobank.org/cytobank/experiments/308354), and non-naïve CD8 T cells (https://premium.cytobank.org/cytobank/experiments/308357). |
| Penttilä PA, et al. [19] | 33715015 | 13-Mar-21 | Human | 40 COVID-19 patients | Whole blood (WB) samples | Immunomodulatory effects |  | - | High-dimensional cytometry by time-of-flight (CyTOF) | No | shows a shift from an initially dysregulated immune response to a more coordinated immunogenic interplay underlying recovery from severe COVID-19. | The datasets generated during the current study are available in the Flow repository, https://flowrepository.org/id/FR-FCM-Z34U. |
| Rendeiro AF, et al. [20] | 33361110 | 24-Dec-20 | Human | 45 COVID-19 patients | Peripheral blood mononuclear cells (PBMCs) | Immune cells |  | - | Flow cytometry | No | depicts a dynamic landscape of immune cells in COVID-19 disease progressions and absolute changes of lymphocyte and myeloid cells with different disease severity. | Hierarchical data format files with single cell data (h5ad) are available as indicated in the repository with source code for the study (https://github.com/ElementoLab/covid-flowcyto). |
| Zhao XN, et al. [21] | 34531370 | 16-Sep-21 | Human | 16 COVID-19 patients | Peripheral blood mononuclear cells (PBMCs) | Immune cells behave | Transcriptomics | Single-cell transcriptome and T-cell/B-cell receptor (TCR/BCR) sequencing | - | No | displays increased CD56briCD16- natural killer (NK) cells, and upregulation of interferon-gamma in effector CD4+ and CD8+ T cells and NK cells, and more robust TCR clonal expansion, but lower interferon-stimulated genes (ISGs) expression with large interpatient variability in asymptomatic patients. | All Raw and processed data are available on CNGB Nucleotide Sequence Archive (CNSA) with accession number CNP0001250. |
| Berentschot JC, et al. [22] | 37881427 | 10-Oct-23 | Human | 37 fatigued and 36 non-fatigued long COVID patients and 42 healthy controls (HCs) | Peripheral blood mononuclear cells (PBMCs) | Immunological profiling |  | - | Flow cytometric analyses | No | provides a point that increased fatigue severity associated with monocyte activation in long COVID-19 patients. | The raw data supporting the conclusions of this article will be made available by the authors, without undue reservation. The Supplementary Material for this article can be found online at: https://www.frontiersin.org/articles/10.3389/fimmu.2023.1254899/full#supplementary-material. |
| Mehta P, et al. [24] | 32192578 | 16-Mar-20 | Human | 125,048 COVID-19 patients | null | Cytokines |  | - | - | No | uses laboratory trends for identification and treatment of hyperinflammation. | null |
| Huang C, et al. [25] | 31986264 | 24-Jan-20 | Human | 41 COVID-19 patients | null | Cytokines |  | - | real-time RT-PCR and next-generation sequencing. | No | Compared with non-ICU patients, ICU patients had higher plasma levels of IL2, IL7, IL10, GSCF, IP10, MCP1, MIP1A, and TNFα. | The data that support the findings of this study are available from the corresponding author on reasonable request.Participant data without names and identifiers will be made available after approval from the corresponding author and National Health Commission. |
| Sekine T, et al. [26] | 32979941 | 14-Aug-20 | Human | 206 COVID-19 patients | Peripheral blood mononuclear cells (PBMCs) | Memory T cells |  | - | Flow cytometry | No | illustate that replete memory T cell responses prevent recurrent episodes of severe COVID-19. | Maximal disease severity was assessed using the NIH Ordinal Scale and Sequential Organ Failure Assessment (SOFA) .Supplemental Information can be found online at https://doi.org/10.1016/j.cell.2020.08.017. |
| Diao B, et al. [27] | 32425950 | 1-May-20 | Human | 522 COVID-19 patients and 40 healthy controls | Peripheral blood mononuclear cells (PBMCs) | T cells and cytokine |  | - | Flow cytometric | No | depicts that T cell counts contribute to the COVID-19 disease. | The raw data supporting the conclusions of this article will be made available by the authors, without undue reservation.The Supplementary Material for this article can be found online at: https://www.frontiersin.org/articles/10.3389/fimmu.2020.00827/full#supplementary-material. |
| Woodruff MC, et al. [28] | 33028979 | 7-Oct-20 | Human | 17 COVID-19 patients | Peripheral blood mononuclear cells (PBMCs) | B cell responses |  | - | High-dimensional flow cytometry | No | provides further evidence that targeted immunomodulatory therapy may be beneficial in specific patient subpopulations. | All FCM data presented are publicly available in the FlowRepository at http://flowrepository.org/id/FR-FCM-Z2XF/. Single-cell V(D) J sequencing is available in the Sequence Read Archive at https://www.ncbi.nlm.nih.gov/bioproject/642962/. |
| Kuri-Cervantes L, et al. [31] | 32669287 | 15-Jul-20 | Human | 42 patients with COVID-19 and 12 healthy controls | Whole blood | Immune cell subsets |  | - | Flow cytometric analyses | No | demonstrates broad innate and adaptive leukocyte perturbations that distinguish dysregulated host responses in severe SARS-CoV-2 infection. | The immunoglobulin heavy chain sequencing data are available in an AIRR-compliant manner to SRA under PRJNA630455. All compensated flow cytometry files are publicly available at https://hpap.pmacs.upenn.edu; please contact MRB for download instructions. |
| Li S,et al. [32] | 33717140 | 24-Feb-21 |  | 10 patients with COVID-19 and 5 health volunteers | Peripheral blood mononuclear cells (PBMCs) | Immune cell phenotype | Transcriptomics | single-cell ATAC (scATAC-seq) and RNA sequencing (scRNA-seq) | - | No | Generated a landscape of chromatin epigenetic status and transcriptomic immune profiles of T cells in patients with COVID-19, and provided a deeper dissection of the characteristics of the T cells involved at a higher resolution than from previously obtained data merely by the scRNA-seq analysis | The raw data reported in this paper have been submitted in the China National GeneBank DataBase (https://db.cngb.org/) and the submission number is CNP0001507. |
| Wilk AJ, et al. [33] | 32514174 | 8-Jun-20 | Human | 7 patients with COVID-19 and 6 healthy controls | Peripheral blood mononuclear cells (PBMCs) | Immune cell phenotype | Transcriptomics | single-cell RNA sequencing (scRNA-seq) | - | No | provide a cell atlas of the peripheral immune response to severe COVID-19. | Processed count matrices with de-identified metadata and embeddings are available for download from the COVID-19 Cell Atlas (https://www.covid19cellatlas.org/#wilk20) hosted by the Wellcome Sanger Institute. Processed data are also available for viewing and exploration on the publicly accessible cellxgene platform by the Chan Zuckerberg Initiative at https://cellxgene.cziscience.com/d/Single_cell_atlas_of_peripheral_immune_response_to_SARS_CoV_2_infection-25.cxg/. Raw sequencing data are available at the NCBI Gene Expression Omnibus (accession no. GSE150728). |
| Zhang Q. et al. [36] | 32972995 | 24-Sep-20 | Human | 1193 patients with COVID-19, including the whole exome (N = 687) or whole genome (N = 506) | Whole blood | Immunity | Genomics | Next-generation sequencing | - | No | reveals essential roles for both the double-stranded RNA sensor TLR3 and type I IFN cell-intrinsic immunity in the control of SARS-CoV-2 infection. | The whole-genome sequencing datasets used for the analyses, including critical patients and asymptomatic controls described in this manuscript, were deposited in dbGaP under accession number phs002245.v1.p1. All other data are available in the manuscript or the supplementary material. |
| Ellinghaus D, et al. [37] | 32558485 | 17-Jun-20 | Human | 1980 patients with COVID-19 and 1255 control participants | Whole-blood samples | Genetic factors | Bioinformatics | - | - | No | identifies a 3p21.31 gene cluster as a genetic susceptibility locus in patients with Covid-19 with respiratory failure. | Genomewide summary statistics of analyses are publicly available through web browser (www.c19-genetics.eu) and have been submitted to the European Bioinformatics Institute (www.ebi.ac.uk/gwas; accession numbers, GCST90000255 and GCST90000256). |
| Wu H, et al. [38] | 35022412 | 12-Jan-22 | Human | 22 patients with COVID-19 | Lung specimens | Immune cells | Transcriptomics | Bulk RNA sequencing and digital spatial profiling (DSP) | - | No | depicts comprehensive high-dimensional transcriptional and spatial immune profiling in severe COVID-19 disease progression. | The RNA-Seq and GeoMx data generated in this study have been deposited in the National Genomics Data Center database under the accession numbers HRA000974,OMIX488 and Gene Expression Omnibus under accession numbers GSE182917, GSE182920. The images can be queried in the database BioStudies under accession number S-BIAD170. |
| Herold T, et al. [39] | 32425269 | 18-May-20 | Human | 89 patients with COVID-19 | null | Cytokines |  | - | - | No | uses IL-6 or CRP level to guide escalation of treatment in patients with COVID-19-related hyperinflammatory syndrome. | Online Repository at www.jacionline.org. |
| Keech C, et al. [42] | 32877576 | 10-Dec-20 | Human | 131 healthy adults | Blood | Immune cells |  | - | ELISA | No | displays the immune responses of the rSARS-CoV-2 vaccine. | <https://www.ncbi.nlm.nih.gov/pmc/articles/PMC7494251/#ap3.> |
| Logunov DY, et al. [45] | 32896291 | 4-Sep-20 | Human | 76 healthy adult volunteers | Blood and urine | Immune cells |  | - | Flow cytometry | No | provides the evidence for cellular immunity after vaccination, and illustrate the effectiveness of this vaccine for prevention of COVID-19. | Individual participant data will be made available on request, directed to the corresponding author (DYL). After approval of a proposal, data can be shared through a secure online platform. |
| Khoury DS, et al. [46] | 34002089 | 17-May-21 | Human | null | null | Neutralizing antibody levels | Bioinformatics | - | - | No | provide an evidence-based model of SARS-CoV-2 immune protection, and aim to bolster memory responses against waning immunity and emerging variants. | All data are freely available from the corresponding author upon request.All code is freely available from https://github.com/InfectionAnalytics/COVID19-ProtectiveThreshold. |
| Korber B, et al. [49] | 32697968 | 3-Jul-20 | Human | 999 patients with COVID-19 | Nose/throat swabs | Tracking Changes in SARS-CoV-2 Spike | Genomics, Bioinformatics | - | - | Yes | global genomic surveillance, informed by immunomics, enables real-time adaptation of vaccine strategies and public health measures to counteract the challenges posed by viral mutations. | Supplemental Information can be found online at https://doi.org/10.1016/j.cell.2020.06.043. |
| Walls AC, et al. [51] | 32155444 | 9-Mar-20 | Animal | null | null | Structure, Function, and Antigenicity of the SARS-CoV-2 Spike Glycoprotein | Bioinformatics | - | - | Yes | depicts the interaction between the SARS-CoV-2 spike protein and the ACE2 receptor on human cells, and provides one critical target for immunotherapeutic interventions. | The cryoEM maps and atomic models have been deposited at the Electron Microscopy Data Bank and the Protein Data Bank with accession codes EMD: 21452 and PDB: 6VXX (closed SARS-CoV-2 S), as well as EMD: 21457 and PDB: 6VYB (SARS-CoV-2 S with one SB open). |
| Gottlieb RL, et al. [54] | 33475701 | 16-Feb-21 | Human | 577 patients with COVID-19 | null | - |  | - | - | No | compared with placebo, treatment with bamlanivimab and etesevimab was associated with a statistically significant reduction in SARS-CoV-2 viral load in nonhospitalized patients with mild to moderate COVID-19 illness. | ClinicalTrials.gov Identifier: NCT04427501. |
| Zhou Q, et al. [56] | 32574262 | 15-May-20 | Human | 77 patients with COVID-19 | Serum | Cytokines |  | - | - | No | IFN-α2b as a therapy in COVID-19 cases. | The raw data supporting the conclusions of this article will be made available by the authors, without undue reservation, to any qualified researcher. The Supplementary Material for this article can be found online at: https://www.frontiersin.org/articles/10.3389/fimmu.2020.01061/full#supplementary-material. |
| Huaman MA, et al. [57] | 37534607 | 3-Aug-23 | Human | nearly 1181 patients with COVID-19 | null | - |  | - | - | No | CCP is a safe therapeutic option for outpatients at risk of hospitalization from COVID-19. | The Early Treatment trial (CSSC-004; NCT04373460) and the Infection Prevention (CSSC-001; NCT04323800) trial. |
| Lam TT, et al. [60] | 32218527 | 26-Mar-20 | Animal | 18 pangolins |  | - | Genomics, Bioinformatics | RNA sequencing | - | Yes | supports the hypothesis that these mammals could be intermediate hosts facilitating transmission to humans. | Data that support the findings of this study have been deposited in the GISAID database (https://www.gisaid.org) with accession numbers EPI_ISL_410538–EPI_ISL_410544 and the SRA database under BioProject accession number PRJNA606875. The data are also available as Supplementary Information. |
| Shi J, et al. [64] | 32269068 | 8-Apr-20 | Environmental, Human, Animal | Dogs, pigs, chickens, ducks, ferrets, and cats | Nasal washes and rectal swabs | - |  | - | RT-PCR | Yes | notes a crucial role in viral replication surveillance in animals in close contact with humans to SARS-CoV-2. | <https://www.science.org/doi/10.1126/science.abb7015.> |
| Palmer MV, et al. [65] | 33692203 | 10-May-21 | Animal | 6 white-tailed deer fawns | Nasal secretions, feces, serum tracheal wash, and lung lavage | - | Bioinformatics | - | - | No | facilitating genomic sequencing to track changes in the viral genome. | <https://www.ncbi.nlm.nih.gov/pmc/articles/PMC8139686/pdf/JVI.00083-21.pdf> |
| Wan Y, et al. [66] | 31996437 | 17-Mar-20 | Human, Animal | null | null | Receptor recognition mechanisms | Bioinformatics | - | - | Yes | provides a predictive framework for novel insights into the receptor usage and likely host range of 2019-nCoV. | GenBank accession numbers are MN908947.1 for 2019-nCoV spike, NC_004718.3 for human SARS-CoV spike (year 2002; strain Tor2), AGZ48818.1 for bat SARS-CoV spike (year 2013; strain Rs3367), AY304486.1 for civet SARS-CoV spike (year 2002; SZ3), and AY525636 for human/civet SARS-CoV spike (year 2003; strain GD03). References for the other sequences are in parentheses as follows: civet SARS-CoV spike (year 2005) (9); human SARS-CoV spike (year 2008). |
| Andersen KG, et al. [70] | 32284615 | 17-Mar-20 | Human, Animal | null | null | The origin of SARS-CoV-2 | Genomics, Bioinformatics | - | - | Yes | Natural selection in an animal host before zoonotic transfer, and in humans following zoonotic transfer. | Sequences shown are from NCBI GenBank, accession codes MN908947, MN996532, AY278741, KY417146 and MK211376. The pangolin coronavirus sequences are a consensus generated from SRR10168377 and SRR10168378 (NCBI BioProject PRJNA573298). |
| Kim YI, et al. [72] | 32259477 | 13-May-20 | Animal | 24 ferrets | Nasal washes, saliva, urine, and feces | Animal model of COVID-19 |  | - | - | No | The ferrets represent an infection and transmission animal model of COVID-19 that may facilitate development of SARS-CoV-2 therapeutics and vaccines. | <https://doi.org/10.1016/j.chom.2020.03.023.> |
| Plante JA, et al. [97] | 33106671 | 26-Oct-20 | Human, Animal | African green monkey kidney epithelial Vero E6 cells and human lung adenocarcinoma epithelial Calu-3 cells | Cell lines | The D614G mutation in the USA-WA1/2020 strain | Bioinformatics | Sanger sequencing | - | No | underscores the importance of D614G mutation in viral spread, vaccine efficacy, and antibody therapy. | Data associated with all figures may be accessed via the Figshare data repository at doi:10.6084/m9.figshare.13030430. |
